# Adsorption Performance Assessment of Agro-Waste-Based Biochar for the Removal of Emerging Pollutants from Municipal WWTP Effluent

**DOI:** 10.3390/molecules30244803

**Published:** 2025-12-17

**Authors:** Dragana Lukić, Vesna Vasić, Jelena Živančev, Igor Antić, Sanja Panić, Mirjana Petronijević, Nataša Đurišić-Mladenović

**Affiliations:** University of Novi Sad, Faculty of Technology Novi Sad, Blvd. Cara Lazara 1, 21000 Novi Sad, Serbia; vesna.vasic@uns.ac.rs (V.V.); jelena.zivancev@tf.uns.ac.rs (J.Ž.); antic@tf.uns.ac.rs (I.A.); sanja.panic@uns.ac.rs (S.P.); petronijevic84mirjana@gmail.com (M.P.); natasa.mladenovic@uns.ac.rs (N.Đ.-M.)

**Keywords:** biochar, agricultural waste, raspberry canes, adsorption, pharmaceuticals, pesticides, contaminants of emerging concern, municipal wastewater

## Abstract

Wastewater treatment plants (WWTPs) have been identified as the major sources of contaminants of emerging concern (CECs) in water bodies, as they are not designed to remove organic micropollutants efficiently. Consequently, many technologies have been explored for WWTP upgrading, including activated carbon adsorption. However, the high production cost and environmental challenges associated with activated carbon production limit its application in industrial settings. Therefore, a wide range of alternative materials has been investigated as potential replacements. In this study, biochar produced from waste raspberry biomass was evaluated as an adsorbent for the removal of pharmaceuticals and pesticides quantified in the secondary effluent of municipal WWTP. The results showed that the biochar efficiently removed almost all detected compounds, except for three compounds (clarithromycin, propranolol, and linuron). The wastewater pH (6–8) did not significantly affect removal efficiency significantly, and kinetic tests demonstrated rapid adsorption. The potential for biochar reuse was confirmed through three consecutive batch adsorption cycles. A comparative study between biochar and powdered activated carbon (PAC) revealed some differences in efficiency, primarily attributed to the larger surface area of PAC. π-π interactions, hydrogen bonding, and pore-filling were proposed as possible adsorption mechanisms based on the adsorption efficiency and biochar characterization.

## 1. Introduction

Pharmaceutically active compounds (PhACs), personal care products, polar pesticides, poly- and perfluoroalkyl substances (PFAS), and industrial chemicals are among the many examples of the wide range of mostly unregulated substances that are commonly found in various environmental compartments, which are referred to as contaminants of emerging concern, CECs [1,2]. The presence of CECs in environmental resources remains a significant challenge due to the excessive production and consumption of these chemicals. Many substances, recognized as CECs, are present in the environment, with concentrations ranging from ng/L to μg/L; in some cases, emissions can be as high as 100 mg/L [3]. Emissions of CECs into environmental resources include various industrial capacities, households, wastewater treatment plants (WWTPs), hospital effluents, direct disposal of unused or expired drugs, manufacturing, landfill leachates, livestock activities, aquaculture, and soil fertilization with sewage sludge and/or livestock waste [4,5,6,7,8,9]. Among these sources, WWTP effluent discharge is considered the main route for CEC distribution into environmental resources [7]. WWTPs have been identified as the main sources of CECs in the environment because they are not designed to efficiently remove organic micropollutants [3,8,9,10]. Accordingly, many CECs survive the conventional wastewater treatment processes [11]. According to Serbian regulations [12], urban WWTP effluents discharged into surface waters are generally characterized by suspended solids, dissolved organic matter, and nutrients, particularly in recipients sensitive to eutrophication. Similar requirements were applied in the European Union until recently. In 2025, the European Parliament adopted a revised version of the Urban Wastewater Treatment Directive [13], which also proposes the removal of CECs by introducing a fourth step (quaternary treatment) in urban wastewater treatment. WWTPs are expected to improve effluent quality, reducing the micropollutant contamination of surface waters and/or enabling water reuse.

Various chemical, physical, and biological treatment techniques have been evaluated for their efficacy in removing these pollutants, but the most commonly investigated are advanced oxidation processes, membrane filtration, and adsorption [14,15].

Since the adoption of the Water Protection Act in 2016 in Switzerland and certain parts of Germany, ozonation and adsorption onto have been employed as hybrid or standalone processes to remove micropollutants in wastewater treatment plants [15,16,17]. The literature provides data on the application of powdered activated carbon (PAC) in biological effluent treatment, but also its direct addition to aeration basins with activated sludge, where the biological degradation of organic matter and adsorption simultaneously occur. The main advantage of this method is that the addition of the adsorbent can be easily implemented into existing aerobic-activated sludge processes, making it more cost-effective compared to a separate treatment stage involving PAC. However, comparative studies of PAC dosing in secondary treatment effluent versus dosing in aeration basins have shown that the latter approach is less efficient and requires almost double the amount of PAC [18]. These approaches have reduced the concentration of a wide range of micropollutants by more than 80% [16].

Adsorption on AC efficiently removes various pollutants from water, including CECs. Still, it is expensive due to high AC production costs and possible nonrenewable resource origins [9,14]. However, this technique is technically feasible and simple [16]. Therefore, low-cost materials, including biochar, have been investigated as substitutes for AC.

Biochar is a carbon-rich material produced from biomass at high temperatures and in a poor oxygen atmosphere in the process known as pyrolysis [19]. Agricultural, forestry, and industrial waste, as well as municipal solid waste, manure, and sewage sludge, can all be used for its production. During pyrolysis, thermal decomposition occurs within a temperature range of 250–900 °C. Depending on the temperature and residence time, pyrolysis can be categorized as slow, fast, or flash pyrolysis [19,20,21,22]. The physicochemical properties of biochar depend on the type of feedstock, the type of pyrolysis, and operating conditions (temperature, heating rate, and residence time) [21,22]. Generally, biochar is characterized by a porous structure, a relatively large specific surface area, and numerous functional groups. Biochars obtained by pyrolysis typically exhibit lower porosity and specific surface area; therefore, they are often subjected to further activation processes to enhance these properties [20]. One way to improve the properties of biochar is the physical activation in a CO_2_ atmosphere at high temperatures. It was found that CO_2_-activated biochar had up to five times higher specific surface area and pore volume compared to before activation, where the properties were highly dependent on the temperature of the activation process [23]. These properties make biochar similar to activated carbon, making it a suitable adsorbent for water and wastewater treatment. The main advantage of biochar is its cost-effective production [19]. Consequently, the potential application of biochar for the removal of various pollutants from water, including CECs, has been widely investigated.

Biochar derived from raspberry canes is a promising but under-explored option for sustainable wastewater treatment. Raspberry is widely cultivated in Western Serbia. Consequently, raspberry canes are generated as waste in large quantities after harvest and are usually burned in fields [24]. According to the data from the Statistical Office of the Republic of Serbia, the areas under raspberry cultivation in 2021, 2022, and 2023 were approximately 20,000 hectares, with a total production of 110,000 tons and yields ranging between 5.2 and 5.9 tons per hectare. Some estimates of the waste biomass generated during raspberry pruning indicate a significant annual output of 1.5–2 tons per hectare [25], which amounts to about 30,000 tons.

Under the concept of a circular economy, the possible application of biochar from raspberry waste addresses waste management issues and transforms agricultural waste into valuable resources. According to the existing literature, the production, characterization, and application of biochar from this type of biomass have not been reported. The main aim of this study was to investigate the properties of biochar obtained from this kind of agricultural waste biomass and its possible application as an adsorbent for CEC removal from municipal wastewater.

To address all these challenges, this study examines the effectiveness of biochar produced from waste raspberry canes for removing contaminants of emerging concern (CECs) from real municipal wastewater. The main objectives were to evaluate its adsorption efficiency toward a broad set of pharmaceuticals and pesticides, compare its performance with commercial powdered activated carbon (PAC), assess the influence of pH and initial pollutant concentration, and determine its reusability over several cycles. Raspberry canes were selected due to their availability as low-value agricultural residue and their suitability for conversion into porous carbon materials.

The adsorption capacity and efficiency of the process depend on many factors, such as the physicochemical properties of the adsorbent and adsorbate, the concentration of the adsorbate, other substances present in the water, and operational conditions. In the case of highly complex and variable matrices, such as wastewater, the efficiency of an adsorbent can only be determined through tests on real wastewater samples. Therefore, all the experiments were conducted on real municipal WWTP effluent. By analyzing biochar application on real wastewater samples, this study provides insights into the simultaneous and complex influence of multiple factors on the adsorption process and the removal rate of CECs by raspberry cane-based biochar.

## 2. Results and Discussion

To identify and quantify CECs in municipal wastewater, sampling was conducted on both the influent and the effluent. It is well known that the quality of wastewater, particularly the influent of the WWTP, varies over time; thus, composite samples provide more reliable data regarding the types of CECs that can be expected in the wastewater. The quality of the effluent also fluctuates over time. However, for further effluent treatment using biochar after the biological process, grab samples are more important as they provide insights into concentrations of individual compounds and their (potentially extreme) variations. Table S1 (Supplementary Materials) summarizes the target compounds investigated in water samples, while Table 1 lists the compounds that were detected, along with their respective concentrations.

According to the literature data, 70% of PhACs in wastewater originate from households, 20% from livestock farming, 5% from hospital wastewater, and the remaining 5% from other sources, with certain variations observed during different seasons [26] and depending on the population socioeconomic composition and the wide range of consumer products used daily [9].

As shown in Table 1 and Table S1, some compounds were not detected in any of the analyzed effluent samples, and some appear sporadically (famotidine, furosemide, losartan, salbutamol, acetamiprid, diazinon, linuron, methamidophos, tebuconazole). Certain compounds (atenolol, carbamazepine, clarithromycin, sotalol), however, were present in all five effluent samples, either at similar or varying concentrations, indicating their nearly constant or variable emission. Literature data suggest that the presence of most compounds listed in Table 1 is characteristic of municipal wastewater [9,26].

### 2.1. Adsorption of CECs by Biochar

The adsorption properties of activated biochar (BC) for removing CECs from WWTP effluent were investigated through batch adsorption to assess the effects of wastewater pH, initial CEC concentration, biochar dose, and time on the adsorption process. Additionally, the feasibility of using biochar through three cycles of multi-step batch adsorption was evaluated, and its efficiency was compared with that of commercial activated carbon. Preliminary tests were conducted to identify the most efficient biochar (either pristine or modified) for additional treatment of WWTP effluent. The results demonstrated that activated biochar is more efficient (Table S2). Thus, all further experiments were focused on activated biochar as an adsorbent.

The Regulation on Limit Values of Emissions of Pollutants in Water and Deadlines for Their Achievement [12] allows a pH range of 6 to 9 for wastewater discharge into surface waters. Effluent samples from WWTP during the sampling period were within the permitted range (8.0–8.4). Therefore, the effect of other pH values within the range on the adsorption efficiency of CECs by biochar was also examined. The results are presented in Table 2.

The obtained activated biochar shows high efficiency for the simultaneous removal of PhACs and pesticides from effluent at different pH values. Within the examined pH range, the minor differences observed were minor and, from a wastewater treatment perspective, not operationally significant. Real effluents vary over time in many parameters (e.g., DOC, ionic strength, competing solutes), and pH is only one of several factors that can influence adsorption. Therefore, small pH-related variations are unlikely to affect overall treatment performance. These results suggest the potential for the direct application of the investigated biochar for the treatment of the WWTP effluent, regardless of pH variations. Literature provides various data on the impact of solution pH on the adsorption of PhACs and pesticides. For example, Fu et al. [27] reported enhanced adsorption capacity of atenolol on corncob-based biochars with increased solution pH from 2 to 9. As the pH increased above 9, the adsorption capacity decreased, indicating that the optimum pH for atenolol removal is 8 and 9, depending on the biochar modification. Naghdi et al. [28] reported an enhancement of carbamazepine adsorption on pine wood nanobiochar as pH increased from 3 to 6. However, authors showed that a further increase in pH from 6 to 8 did not affect the adsorption process, which is consistent with the results presented in Table 2. Similar results were reported by Chu et al. [29]. Namely, the adsorption of carbamazepine onto non-modified and bleached biochar from pine sawdust did not vary in the pH range of 4–8. Within the solution pH range 3–9, carbonized coconut shell-based biochars modified with H_3_PO_4_ and NaOH showed the highest removal of diazinon at pH 7 (over 85%). At the same time, only pyrolyzed material was more efficient in diazinon removal at pH 3, reaching 92% [30]. Moussavi et al. [31] also reported the best diazinon removal at neutral pH on NH_4_Cl-induced activated carbon. The adsorption capacity of tebuconazole on waste-activated sludge-based biochar dropped slightly as the solution pH increased from 3 to 10, from 12.4 to 11.4 mg/g [32]. On the other hand, pH did not affect the adsorption of linuron across the same range [32]. Similarly, Moraes et al. [33] reported a lower efficiency of linuron adsorption on commercially available activated carbon, and ZnCl_2_ and HNO_3_ spent coffee grounds-based activated carbons (~45% for all the adsorbents) over a wide pH range. However, the reported data were obtained in synthetic solutions, so different results can be expected in much more complex matrices such as real wastewater.

The adsorption capacity of the biochar and the CEC removal depend on the concentration and type of pollutants and the coexistence of various contaminants [34], which is a common case in wastewater. Dissolved organic matter can negatively affect CEC adsorption due to competition for active sites and the potential to block micro- and mesopores, given their molecular weights range from several hundred daltons (Da) to over 100 kDa. Compounds with lower molecular weights may have a greater impact on CEC adsorption [35]. The content of organic matter varies in both wastewater and treated water, depending on the type of treatment applied. According to the average annual values (Section 3.3.1 in Section 3 Materials and Methods), the content of organic matter (expressed as COD and BOD_5_) in the effluent from the biological treatment at the WWTP falls within a very narrow range, suggesting that significant deviations in the efficiency of CEC removal by biochar shown in Table 3 are not expected, regardless of change in CEC concentrations in a reasonable range.

Numerous emerging contaminants in wastewater differ significantly in their physicochemical properties (including molecular structure, hydrophobicity, aromaticity, and polarity). As a result, their sorption onto biochar surfaces may arise from synergistic effects or competition among multiple compounds [34]. The quality of municipal wastewater, in terms of the type and concentration of these compounds, varies depending on the size of the settlement, lifestyle, population habits, and the time of year. To examine the efficiency and capacity of biochar at higher concentrations of CECs, the effluent was further spiked with the quantified compounds (Table 4). Furthermore, other selected compounds that had not been previously detected in the effluent (known to be quantifiable in WWTP effluents according to the previous studies) were spiked into the effluent. Spiked CEC concentrations of the effluent were chosen based on literature data, i.e., concentrations characteristic of wastewater in the Western Balkans region during the period from 2008 to 2023 [9]. The results are presented in Table 4.

According to the data presented in Table 3 and Table 4, biochar is highly effective in removing CECs across a wide range of environmentally relevant concentrations. Only bezafibrate and diltiazem out of all compounds were removed with an efficiency of less than 91% at higher concentrations. This is in agreement with the reported results of Moraes et al. [33]. Namely, the lower efficiency of linuron adsorption on commercially available activated carbon and spent coffee grounds-based activated carbons (~45% for all the adsorbents) was noted. The removal efficiency of raspberry biochar did not decrease even when the effluent was spiked with additional compounds due to a sufficiently large surface area and active sites. Moreover, the removal of propranolol is much higher at higher concentrations.

An adsorption study of carbamazepine on spent brewery grains-based biochars showed that the adsorption capacity was significantly lower in a synthetic solution compared to real wastewater. It was assumed that some inorganic and organic matter present in wastewater can affect adsorption by competition for active sites or block access to the pores on the adsorbents [36]. Diniz et al. [37] also reported a decrease in the adsorption capacity of different CECs in wastewater compared to synthetic solutions up to 50% despite higher doses of commercially available activated carbon. These results imply that even if the adsorption is affected by a complex matrix, removal of various CECs is possible by adsorption. However, tests in real wastewater are only valid for predicting the performance of adsorbents during their actual application.

**Figure 1:**
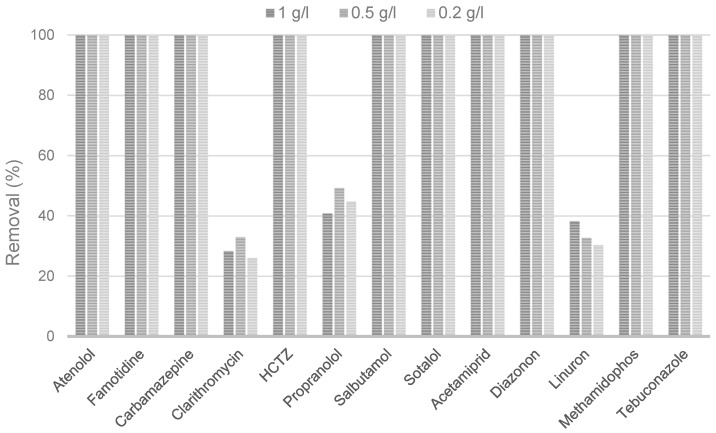
Removal efficiencies of CECs depending on the applied biochar dose.

The dose of biochar is a crucial parameter influencing the quality of the treated water and the overall cost of the adsorption process. The optimal dose depends on the type of adsorbent, wastewater composition, and the required pollutant removal. Usually, it is determined by laboratory testing or based on previous experience, and it is confirmed during the initial implementation phase at the industrial scale. At high concentration levels of pollutants, increasing the dose of biochar can significantly enhance the removal due to the larger overall specific surface area and the greater number of active sites. Nevertheless, above a certain dose, biochar may form agglomerates, resulting in a reduced total surface area and thus decreased efficiency. This phenomenon is more likely to occur with biochars produced from materials with a high lignin content [34]. Moreover, the higher the lignin content, the higher the yield and fixed carbon in biochar that may assist the adsorption of organic contaminants [38]. The lignin content in lignocellulosic materials is typically up to 35% [39]. In raspberry canes, it is relatively high, exceeding 30%, depending on the particle size [24], but not as high as in coconut shells (around 50%) [40]. The efficiency of biochar at different doses was investigated, and the results are presented in Figure 1.

Results showed that the lowest investigated dose (0.2 g/L) may be sufficient for the efficient removal of CECs from WWTP effluent and that the removal efficiency does not decrease with a decrease in biochar dose from 1 to 0.2 g/L. The doses applied in this study are somewhat higher than typical doses of commercial activated carbon used in water and wastewater treatment. However, they are in the range of biochar doses reported in the literature for organic pollutants removal [41,42,43,44]. Naghdi et al. [28] reported an increased removal efficiency of carbamazepine from 53% to 87% with the increase in pinewood-based biochar dose of 0.2–1 g/L. Higher doses did not show any improvement. Biochar produced from pineapple leaf non-fibrous material showed an acetamiprid removal efficiency of 84.3% at 5 g of adsorbent per liter of solution and negligible removal increase at 7 g/L, suggesting biochar saturation [45]. Yang et al. [32] reported that the efficient removal of tebuconazole (87.8%) and linuron (95%) from a single-component solution was achieved with 1.25 g/L of waste-activated sludge-based biochar modified with FeCl_3_.

**Figure 2:**
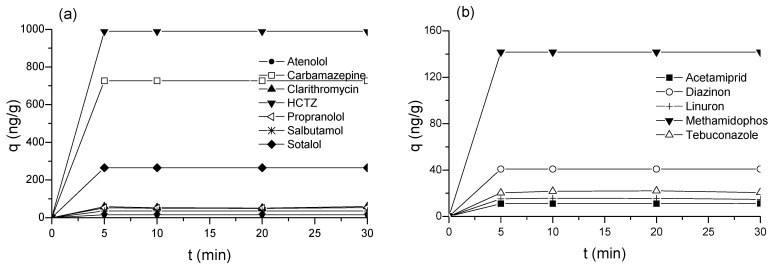
Adsorption kinetics of: (**a**) PhACs; (**b**) Pesticides on biochar from WWTP effluent.

Adsorption is a time-dependent process, and for performance optimization, it is crucial to determine the time required for the process to reach equilibrium. For the practical application of an adsorbent, its adsorption capacity is equally important as the reasonable period necessary for the process to end. Adsorption kinetics of PhACs and pesticides are shown in Figure 2.

Adsorption of all CECs found in WWTP effluent onto biochar was very rapid. All detected compounds reached equilibrium within the first 5 min of contact. Literature reports much longer contact times between biochar and wastewater. Coimbra et al. [46] showed that the adsorption of selected pharmaceuticals onto pyrolyzed pulp mill sludge reached equilibrium within 200 min from a solution and secondary municipal WWTP effluent. Silva et al. [47] conducted an adsorption study of selected pharmaceuticals on laboratory-produced activated carbon and commercial activated carbon from a solution and biological treatment effluent of municipal wastewater. Adsorption kinetics experiments showed that it took 60–240 min for all the compounds to reach equilibrium on both adsorbents in the mixture and wastewater. Fast adsorption greatly improves the economic efficiency of the process, allowing the use of smaller reactors and lower doses of adsorbent. Short contact times, ranging from 18 to 30 min [48,49] to 0.7 to 3 h [50], are enough for PAC applications in wastewater treatment.

**Figure 3:**
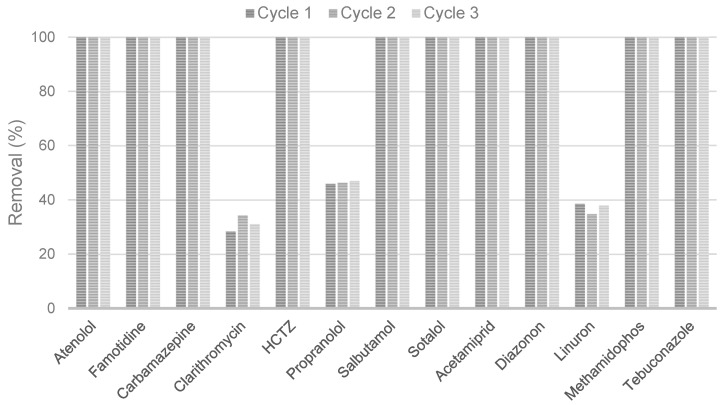
Removal efficiencies of CECs in three consecutive batch adsorption cycles (dose 1 g/L, contact time 45 min).

When PAC is used for wastewater treatment, in some cases, the contact time or the concentration of pollutants could be insufficient to reach the adsorption capacity, resulting in an unsaturated adsorbent. For more effective exploitation, batch adsorption can be conducted as a multistage process with a countercurrent flow of untreated water and PAC. Namely, after the initial use, the spent PAC is separated from the treated water and reintroduced to the new amount of fresh, untreated water. This can significantly reduce the overall demand for PAC for wastewater treatment. As previously mentioned, a relatively short contact time is typically sufficient when PAC is used in a batch mode. Given the very short contact time required to complete the adsorption of detected CECs from the effluent onto biochar (Figure 2), a contact time of 45 min was chosen for the multistage adsorption tests. Three cycles were conducted, and the results are presented in Figure 3.

Almost all the compounds found in the effluent, under the applied conditions, are removed with maximum efficiency across all three stages of the multi-step adsorption, which is attributed to the sufficiently large total adsorption capacity of the biochar (Table 4). However, the removal efficiencies of clarithromycin, propranolol, and linuron were lower but within the characteristic range for this adsorbent/adsorbate system. To provide stronger evidence of adsorption stability, the adsorption capacities (q, ng/g) were calculated for each cycle. The values showed random variation across the three cycles: propranolol 35.9–36.34 ng/g, clarithromycin 21.4–25.8 ng/g, and linuron 26–34 ng/g. These results confirm that the adsorption sites remain largely available and the biochar retains its adsorption potential through the three consecutive cycles. Therefore, the adsorption efficiency of CECs by biochar from the biological treatment effluent does not decrease through these three cycles of multi-step batch adsorption, indicating the potential for its further use until saturation.

### 2.2. Comparative Studies of Biochar and PAC

Given that biochar was produced as a potential alternative to activated carbon in wastewater treatment for reducing CECs emissions into surface waters, comparative studies were conducted on the efficiency of biochar and commercial PAC. The results are presented in Table 5. Based on the results, it can be concluded that both biochar and PAC are efficient in removing CECs detected in the wastewater from the WWTP. It should be noted that these results are not intended to provide a full performance comparison of these two adsorbents, but rather to illustrate their removal efficiency under the specific experimental conditions relevant to the requirements of the new EU Directive on urban wastewater treatment.

A significant difference was observed in the sorption of salbutamol and linuron. Their removal by PAC was about 45% and 100% (45 min contact time), respectively. At the same time, biochar achieved complete removal of salbutamol, whereas linuron was less efficiently removed by biochar. These differences should be interpreted with caution, since the adsorbents were tested on different effluent samples. Although all other experimental parameters were identical, the variability of WWTP effluent composition (e.g., DOC concentration, competing organic matter, etc.) can influence removal efficiencies. Mailler et al. [51] tested 4 commercialized activated carbons of different origins recognized as efficient for organic pollutant removal. Wood-based activated carbon PB170^®^ showed the best removal efficiency for all investigated compounds from WWTP effluent at a typical dose of 10 mg/L. The removal after 45 min was 54% for atenolol, 63% for carbamazepine, and 81% for propranolol. The average removal for 15 different compounds was 52%. The concentrations were in the range common for treated wastewater. The removal was in accordance with specific surface and pore volume—the most efficient adsorbent had the largest surface area and pore volume.

PAC is known as an efficient adsorbent for a wide range of CECs. However, removal largely depends on the applied dose. Literature data show that an average removal of 80% is achieved with PAC doses ranging from 7 to 20 mg/L, depending on the concentration of dissolved organic carbon (DOC) in the wastewater [14]. Taking this into account and the high removal efficiency presented in this study, a lower dose of biochar (20 mg/L) was also tested. A comparative study of PAC efficiency at the same dose was also conducted. However, in both cases, it was insufficiently efficient for CEC removal (Table S3). This further confirms that the applied dose is a key factor influencing CEC removal, and that our aim was not to show that one adsorbent performs better than the other. The intention was to demonstrate that biochar can perform equally well or, at least, comparably to the commercially available adsorbents.

The removal efficiencies of various biochars of CECs in the synthetic solutions are presented in Table 6. Results show their high efficiency and significantly slower adsorption for most of the components compared to raspberry biochar. However, all of these studies are conducted in synthetic single-component solutions or mixtures that are less complex matrices compared to real wastewater. Thus, their efficiencies in real wastewater are likely to be lower.

### 2.3. Biochar Characterization

#### 2.3.1. Yield of Biochar

Raspberry canes, like any lignocellulosic biomass, are a complex mixture of hemicellulose, cellulose, lignin, and small amounts of other organic molecules. Each component is characterized by different degradation kinetics during pyrolysis depending on the process parameters—biomass type, temperature, heating rate, and retention time. Lignin decomposes over a wider temperature range than cellulose and hemicellulose [53]. The biomass type, temperature, particle size, and heating rate essentially determine the physicochemical properties of the obtained biochar. These parameters directly influence the quality of biochar, while its yield is affected by the type and conditions of pyrolysis [54]. Generally, the yield of biochar produced through slow pyrolysis at lower temperatures is higher than that produced through fast pyrolysis at higher temperatures [55]. The obtained biochar yield after the pyrolysis of raspberry cane was 25%, and after a 1 h activation time, the activated sample was produced at a decreased yield of 15%. The applied activation process resulted in a lower yield of biochar compared to the non-activated sample, which is a consequence of the prolonged exposure of the sample to a higher temperature, as well as the presence of oxygen originating from the CO_2_ gas stream and supporting the subsequent decomposition of organic matter [56]. Similar yields were reported for biochars produced from wood biomass (around 20%), and the yields of biochars activated by physical methods were even less (2–17%), depending on the pyrolysis type and its operational conditions [17].

#### 2.3.2. Characterization of Biochar and PAC

High-resolution scanning electron microscopy (HRSEM) coupled with X-ray energy dispersion (EDX) method was applied to determine the surface morphology and elemental composition of biochar and PAC samples. Several dots and representative regions were selected and analyzed for each sample.

**Figure 4:**
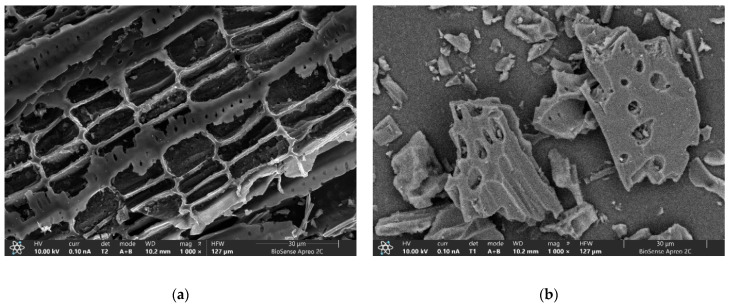
HRSEM images (magnification: 1000×) of: (**a**) Biochar; (**b**) PAC.

As can be seen from Figure 4, both samples have a characteristic heterogeneous surface and a developed macroporous structure. Their morphology shows a resemblance to the original lignocellulosic cell wall structure characterized by the presence of layered structures in combination with channel-like structural units with sharp edges. These morphological shapes are portrayed by exceptionally developed macroporous structures in the shape of a honeycomb with cavities of large diameters in the range 1–10 μm on PAC, and 1–30 μm on biochar. The HRSEM analysis indicates that the remaining pores found in both samples belong to mesopores (diameters up to 50 nm according to IUPAC nomenclature) and were additionally determined by the gas physisorption method.

**Figure 5:**
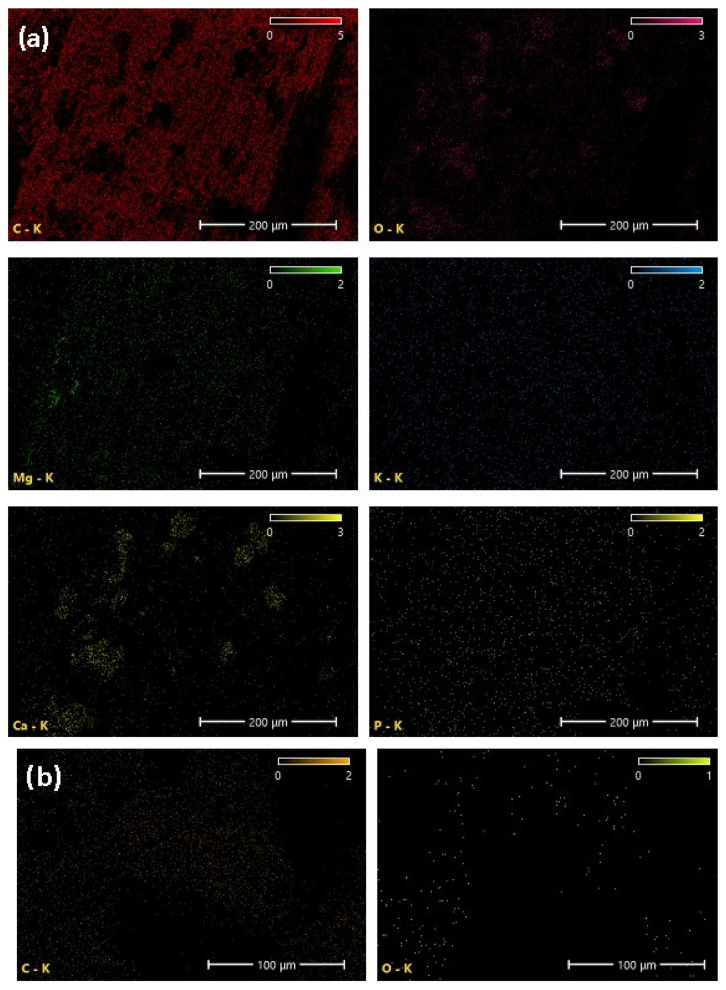
EDX element mapping for: (**a**) Biochar; (**b**) PAC.

The elemental composition (wt%) of biochar and PAC obtained by EDX is shown in Table 7, while the performed EDX mapping with the distribution of the present elements is displayed in Figure 5.

Biochar contains less carbon (60.9 wt%) than the PAC (97.9 wt%) since its elemental composition is more scattered with the presence of trace elements. In general, the chemical composition of biochar is very heterogeneous and depends on the type of biomass, location, and cultivation conditions, as well as the method for biochar production. The most abundant elements in biochar are carbon and oxygen, while other elements, such as Mg, P, K, and Ca, are present in smaller amounts. These elements, as mineral nutrients usually present in plants, are also found in the composition of biochar as a product of raspberry cane pyrolysis. The EDX mapping of PAC showed the homogeneous distribution of carbon and scattered distribution of oxygen, while the biochar is characterized by the homogeneous distribution of C, Mg, K, and P. The oxygen and calcium are mainly dispersed in the form of small islands, i.e., regions of squeezed distribution.

**Figure 6:**
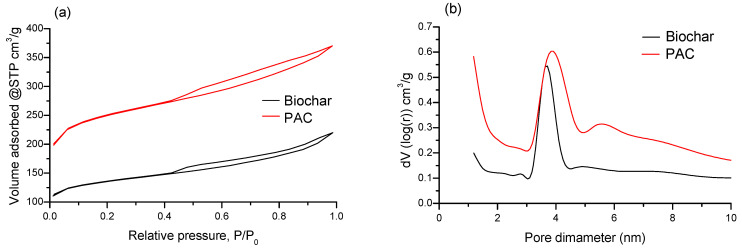
(**a**) Adsorption–Desorption isotherms (hysteresis); (**b**) pore size distribution for biochar and PAC.

N_2_ adsorption–desorption isotherms and BJH pore-size distributions of the activated biochar and PAC are presented in Figure 6. The adsorption–desorption isotherms (Figure 6a) of both samples exhibit an H4-type hysteresis loop, indicative of a predominant presence of micropores in the form of narrow cracks [57]. Both samples show similar pore size distributions, with the majority of pores located in the micropore range (diameter < 2 nm), while mesopores (2–50 nm) display a monomodal distribution (Figure 6b). The specific surface area was determined using the BET method within the relative pressure (P/P_0_) range of 0.05–0.30. Micropore surface area and volume were estimated using the t-plot method.

PAC exhibits a significantly higher specific surface area (829.01 m^2^/g) than biochar (450.05 m^2^/g), due to a larger fraction of micropores (0.32 cm^3^/g). The total pore volume of PAC, including pores smaller than 32 nm, is 0.55 cm^3^/g, indicating that approximately 60% of its pores are micropores, with the micropore surface area accounting for 650.87 m^2^/g (≈80% of the total specific surface area). Biochar shows a lower total pore volume (0.31 cm^3^/g) with a micropore volume of 0.16 cm^3^/g (≈50%) and a micropore surface area of 341.93 m^2^/g (≈75%). These results demonstrate that PAC has a higher degree of microporosity compared to biochar, highlighting that the activation process preferentially enhances microporosity over mesoporosity in these two samples.

**Figure 7:**
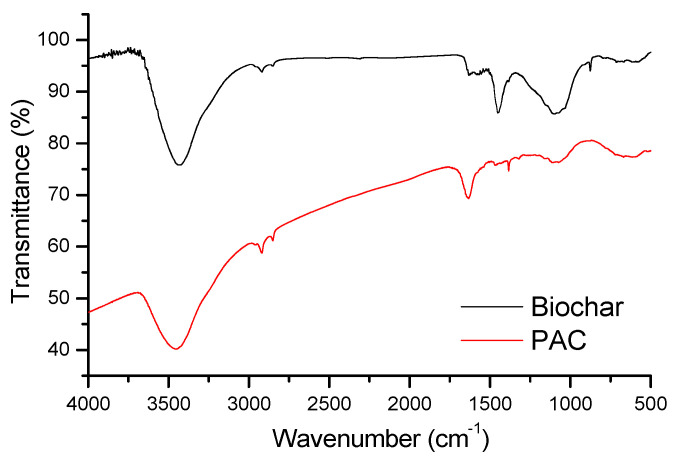
FTIR absorption spectra of biochar and PAC.

The feedstock biomass and the pyrolysis temperature are the most significant factors influencing the functional groups on the biochar surface. When biochar characteristics, such as pH, total surface area, and porosity, are high, the content of these functional groups is generally lower [20]. To identify functional groups on the surface of the activated biochar and PAC, FTIR analyses were conducted (Figure 7).

Biochar is characterized by a broad and intensive vibrational peak around 3420 cm^−1^ assigned to the stretching vibrations of carboxylic and phenol –OH groups, while the –CH_3_ and –CH_2_ stretching vibrations (C–H) are visible in the range 2800–3000 cm^−1^. The band at 1630 cm^−1^ corresponds to the carbonyl functionality [58]. The range of wavenumbers between 1610 and 1475 cm^−1^ includes multiple peaks of very low intensities characteristic of the –C=C– stretching of aromatic components, –C=O stretching of conjugated ketones and quinones, as well as the –C=C– stretching indicative of lignin and aromatic C [59]. The presence of carbonate ions is revealed by the sharp and highly intensive band areas of 1452 and 876 cm^−1^ attributed to their asymmetric stretching vibrations and bending vibrations out of the plane. The wide and highly intensive peak around 1100 cm^−1^ was also identified in the FTIR spectra, demonstrating that various superficial oxygen-containing groups formed carbon-oxygen single bonds with adjacent carbons (–C–O–C– symmetric stretching) [58]. The FTIR spectrum of PAC is not significantly different in comparison with the biochar FTIR spectrum. The same peaks are visible around 3400 cm^−1^, in the area of 2800–3000 cm^−1^, around 1630 cm^−1^, and 1640 cm^−1^. The peak visible at 1384 cm^−1^ is assigned to C=C groups of the aromatic rings [60], and 1110 cm^−1^ to –OH stretching in COH group [61].

To know surface chemistry, the pH of dispersion of biochar was determined since it is an important characteristic affecting adsorption and depends on the solution pH and surface functional groups. Namely, higher pyrolysis temperatures produce alkaline biochar due to the loss of acidic groups (thus, a larger amount of total basic groups) and higher content of alkali and alkaline earth metals in ash [36,62]. The raspberry cane-based biochar is alkaline with a pH value of 10.8.

### 2.4. Adsorption Mechanism of CECs

Considering all the factors affecting the adsorption process and the diverse properties of PhACs and pesticides (molecular structure, hydrophobicity, aromaticity, and polarity), it is clear that the mechanism of adsorption is a complex process that includes different interactions such as pore-filling, hydrogen bonding, π-π electron donor-acceptor interactions, hydrophobic, and electrostatic interactions, etc. [34,38]. Understanding these interactions in complex matrices would help manage wastewater treatment processes and develop efficient methods for their removal.

The molecular size of the examined CECs (Table S4) and the pore structure of the biochar and PAC represent very important parameters affecting the adsorption capacity since molecules with smaller dimensions can easily penetrate the adsorbent pores. According to the results of textural characterization, both adsorbents are predominantly microporous materials with similar values of average pore diameter and share of micropore volume. A significantly higher specific surface area obtained for PAC is related to its higher total pore volume. Implying that all the tested molecules of CECs are sufficiently small to access the pores (e.g., the maximum size of carbamazepine is 1.17 nm [63]), the higher adsorption capacity of PAC for some CECs, compared to biochar, could be attributed to its higher specific surface area and volume of micropores. However, since the initial concentrations of all identified CECs in the real wastewater sample are quite low, the previously mentioned difference is likely to be the consequence of other possible adsorption mechanism scenarios.

Hydrophobic interaction is another possible adsorption mechanism occurring due to the tendency of non-polar groups of CECs to aggregate in aqueous solution and therefore, minimize their contact with water molecules. The CECs identified in the real wastewater sample have different hydrophobicity, with logK_ow_ values for PhACs from −0.07 for HCTZ to 3.48 for propranolol, and pesticides from 0.80 for acetamiprid to 3.81 for diazinon (Table S4). In general, compounds with a higher value of logK_ow_ tend to adsorb better on the surface of carbon-based materials due to their more hydrophobic nature. However, adsorption test results showed that both biochar and PAC are very effective adsorbents for highly polar CECs, as well as for the more hydrophobic ones, possibly due to the contribution of the different densities of polar moieties on their surface. The elemental composition of the CO_2_-activated biochar (higher C content and lower O/C ratio) suggests a moderately hydrophobic carbon framework, which can still contribute to the adsorption of neutral or non-polar species. Considering the obtained removal efficiencies of CECs by both adsorbents, a direct correlation between them and the appropriate logK_ow_ values could not be determined, suggesting that these interactions are not the ones controlling the adsorption process.

During the adsorption process, electrostatic interactions may occur between ionizable functional groups of CECs and ionizable functional groups present on the surface of biochar and PAC. The raspberry cane-based biochar is alkaline with a pH value of 10.8, while the PAC is slightly acidic with a pH value of 5.02. The pH of the solution is a very important parameter determining the surface charge of sorbents and the degree of dissociation of the PhAC and pesticides [34]. The pH value of the examined real wastewater sample was around 8. Under these conditions, the number of protonated surface groups on the alkaline biochar is expected to be relatively low, while the more acidic PAC surface generally carries a higher proportion of protonated functionalities at this pH. According to the literature data (Table S4), the majority of the tested PhACs have pK_a_ values above the pH of the solution, suggesting that their molecules mainly exist in neutral form in the wastewater sample [64]. For example, the pK_a_ of atenolol is 9.6. A significant part of atenolol is positively charged at low pH, but as pH increases above 4, molecules become more neutral [27]. Thus, at the pH of WWTP effluent (6-8), electrostatic interactions between biochar and atenolol are insignificant. The same conclusions regarding electrostatic interactions between raspberry biochar and carbamazepine, clarithromycin, propranolol, ranitidine, salbutamol, sotalol, and linuron could be drawn. However, almost all the compounds were removed successfully, suggesting that some other mechanisms involved in adsorption are dominant. The lower adsorption efficiency of clarithromycin can be explained by repulsive forces, but also by the fact that it is a larger molecule compared to other components, which facilitates diffusion into pores. The comparative study of biochar and PAC efficiency showed better clarithromycin removal by PAC, probably due to a larger surface area and average pore diameter obtained for the measuring range (1–100 nm). The same assumption regarding electrostatic interactions can be made in the case of pesticides, diazinon and tebuconazole, with pK_a_ values below the pH of the solution, 2.6 (Table S4) and 5.03 [65], respectively. Both compounds, possibly carrying a negative charge, were completely removed by biochar and PAC, implying that the obtained adsorption capacities are not caused by electrostatic interactions. In addition, Moussavi et al. [31] reported that NaCl as a background electrolyte and ammonia slightly improved the diazinon adsorption, probably due to the compression of the diazinon electrical double layer, and the formation of a complex diazinon-ammonia that adsorbed on activated carbon, respectively [31]. Since the adsorption of diazinon in this study was conducted in real wastewater, the effect of these pollutants could contribute to the removal efficiency. The most significant differences in the adsorption capacity of the examined adsorbents were obtained in the case of acetamiprid and linuron. The pK_a_ of acetamiprid is 0.7 (Table S4), which is far below the pH of the wastewater sample, indicating the possible existence of its negatively charged molecules. However, considering the chemical structure, acetamiprid molecules do not contain functional groups that can be deprotonated, referring to their probable neutral form under the given experimental conditions and insignificant contribution of electrostatic interactions in the adsorption mechanism. Linuron, with a pK_a_ value of 12.3 (Table S4), has nitrogen atoms in the urea group as potential protonatable sites on the molecule. Since the higher degree of protonation would require more acidic conditions (pH < 7), at pH 8, linuron is more likely to exist in its neutral, deprotonated state. In the case that a small proportion of protonated species exists, it could be adsorbed by the negatively charged PAC and electrostatically repulsed by the positively charged surface of biochar.

Two types of hydrogen bonding interactions, as another possible adsorption mechanism, can occur between the examined molecules of CECs and the surface of biochar and PAC. The FTIR spectra confirmed the presence of hydroxyl groups at the surface of the sorbents which can act as H-donors, while the oxygen and nitrogen atoms of the PhACs and pesticides can act as H-acceptors, resulting in dipole–dipole hydrogen bonding [66]. Considering the chemical structure of all CECs, it can be suggested that this type of interaction is significant during the adsorption process since all the molecules have oxygen- and/or nitrogen-containing species that could be involved in dipole–dipole formation (carbonyl, hydroxyl, ester, amino, imino, nitrile groups, etc.). Another type of H-bonding, Yoshida type, occurring between the hydroxyl groups present at the surface of the sorbents and aromatic rings of the adsorbate [67] could considerably enhance the adsorption affinity of both adsorbents. Given the abundance of aromatic structures in tested CECs (benzene rings, as well as nitrogen- and sulfur-containing fused heterocycles), it is plausible that these interactions play an important role in their adsorption. The only exception is related to the clarithromycin molecule. Due to the absence of its aromatic character, its removal efficiency is decreased on both adsorbents. For example, a predominantly neutral carbamazepine can form hydrogen bonds between carbamazepine –NH_2_ and oxygen functional groups present at biochar surfaces, such as –OH and C=O [28]. More efficient adsorption of carbamazepine could be expected at higher pH due to lower concentrations of H^+^ ions when its hydrogen donor groups can interact with hydrogen bonding acceptors or P donors in pine wood nano biochar [28]. FTIR spectrum of raspberry biochar confirmed the presence of relevant functional groups, so the high carbamazepine removal efficiencies obtained in this study in the pH range of 6–8 could be explained by the same mechanism. Atenolol also has –NH_2_ and –OH groups; thus, can adsorb onto biochar surface by the interaction of dipole moment with –OH groups on biochar detected by FTIR [68]. The aromatic structure of both sorbents and almost all tested CECs (except clarithromycin) might indicate that π-π interactions tend to exert a dominant influence, occurring between the aromatic rings of CECs and the aromatic structure of graphene layers of the biochar and PAC. This is supported by the FTIR evidence of C=C structure. Besides these interactions, n-π interactions can also take place between surface oxygen groups in sorbents (−COOH and –OH), acting as electron donors, and aromatic rings of CECs molecules acting as electron acceptors [69]. The investigated pH range (6–8) showed no influence on HCTZ adsorption on raspberry biochar, and it was reduced below the quantification limit of the analytical method. Since HCTZ was predominantly neutral under these conditions, the suggested adsorption mechanisms are π-π interactions and hydrogen bonding. Similar findings were reported by Diniz et al. [37]. However, the share of this type of interaction in the overall mechanism for each tested pollutant depends on the amount of surface oxygen-containing groups present on the surface of the biochar and PAC.

**Figure 8:**
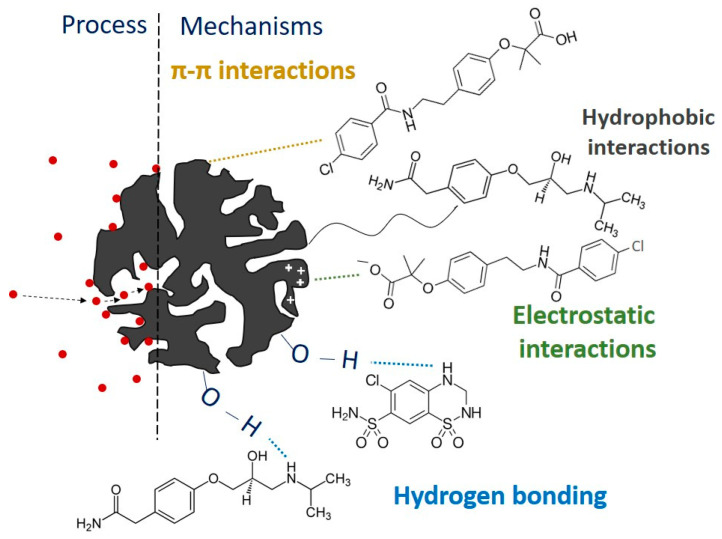
Proposed mechanisms of adsorption.

Based on the previous discussion, it can be suggested that the prevailing microporous structure of the biochar and PAC provides abundant adsorption sites, but, like electrostatic and hydrophobic interactions, is not the determining reason for the high adsorption capacity. Hydrogen bonding, as well as π-π and n-π interactions, appear to be the controlling mechanisms providing strong sorption affinity of biochar and PAC for the tested PhACs and pesticides (Figure 8).

## 3. Materials and Methods

### 3.1. Synthesis and Activation of Biochar

Raspberry canes were air-dried, ground, and sieved through a vibrating screen to isolate the particle size fraction of 1.03–2.00 mm, which was used as the feedstock for biochar production. Biochar was synthesized via slow pyrolysis, defined as a thermochemical decomposition of lignocellulosic biomass at moderate heating rates (typically <20 °C/min) and relatively long residence times under an inert atmosphere [70,71]. Biochar was synthesized in a horizontal fixed-bed quartz reactor following the temperature program described below.

A mass of 4 g of the pre-processed raspberry cane material was placed into a quartz sample holder. To ensure an oxygen-free atmosphere, the pyrolysis reactor was flushed with nitrogen for 10 min before heating. The system was then heated to 700 °C under a continuous N_2_ flow (110 mL/min) at a heating rate of 10 °C/min, including a holding period of 15 min at 350 °C. The residence time at 700 °C was 2 h. After the pyrolysis step, the reactor was cooled to room temperature under an N_2_ flow (110 mL/min). The obtained biochar was subsequently subjected to physical activation under a CO_2_ flow (110 mL/min). The sample was heated at 10 °C/min up to 800 °C and held for 1 h (without an additional holding period at intermediate temperatures). Following activation, the reactor was cooled to room temperature under N_2_ flow (110 mL/min), after which the biochar sample was removed and weighed. To verify that no background mass loss originated from the reactor system itself, a blank run was performed under identical heating and gas-flow conditions using an empty quartz sample holder. No measurable mass change was observed. Thus, all recorded mass losses during pyrolysis and activation were attributed exclusively to biomass decomposition. A mass balance check was carried out by measuring the mass of the biomass before pyrolysis (m_0_) and the mass of the resulting biochar after each step (m). These values were used to calculate the biochar yield according to Equation (1).

Y(%) = m/m_0_·100 (1)

### 3.2. Adsorbent Characterization

We assessed the morphology and elemental composition of the biochar by using a High-Resolution Scanning Electron Microscope (HRSEM), Thermo Fisher Scientific Apreo 2C (Waltham, MA, USA). Fourier Transform Infra-Red Spectroscopy (FTIR) analysis was performed on a BRUKER Vertex 70 IR spectrometer (wavenumber range of 400–4000 cm^−1^ with a resolution of 2 cm^−1^) (BRUKER, Billerica, MA, USA) on a solid sample prepared by the KBr method. The Brunauer–Emmett–Teller specific surface analyses were conducted using an Anton Paar NOVAtouch LX2 instrument (Graz, Austria) after degassing materials for 6 h at 110 °C in a vacuum. Brunauer–Emmett–Teller (BET) specific surface area of materials was calculated from the adsorption branch, in the relative pressure range from 0.05 to 0.3 P/P_0_. Barrett, Joyner, and Halenda (BJH) calculations of pore diameter were performed on the desorption branch of the isotherm.

Bichar pH was measured after mixing 0.5 g of biochar in 50 mL of deionized water for 1 h and allowing it to stand for another 30 min [72].

### 3.3. Adsorption Experiments

#### 3.3.1. Wastewater

We performed all adsorption experiments on a real wastewater sample, i.e., effluent of biological treatment from an urban WWTP. The wastewater treatment process includes preliminary (mechanical treatment: coarse and fine screening, grit removal), primary sedimentation, biological treatment (removal of biodegradable organic matter and nutrients), and subsequent reduction of total phosphorus and suspended solids using a membrane process (with the addition of coagulants) before discharge into the receiving body.

Table 8 presents the average annual values of general quality parameters of treated WWTP effluent at the discharge point into the receiving water body. The quality of the effluent meets the requirements of the Serbian Regulation on Emission Limit Values for Pollutants in Water and Deadlines for Their Achievement [12] for the discharge of treated municipal wastewater into the receiving body. However, it is to be expected that soon, Serbia will have to adopt EU regulations as an EU candidate, including the revised Urban Wastewater Directive.

Wastewater sampling was conducted on the influent as a 24-h composite sample for CEC analysis. We collected effluent samples as grab samples (50 L per campaign) of the secondary treatment effluent in the period March-June 2024. Before each set of experiments, wastewater sampling was conducted (campaigns I–V) due to variations in the wastewater composition and the fact that certain CECs go through biotic and abiotic degradation processes over time. Consequently, the number of quantified compounds and their concentrations varied across different investigations. For this reason, the initial concentrations of CECs in the effluent were determined for each experimental set, and removal efficiency was calculated based on these initial concentrations.

#### 3.3.2. Batch Adsorption

We assessed the adsorption performance of the biochar in the batch mode with two replicates. All the results are presented as mean values. For the kinetic study, 1 g of biochar was agitated with 1 L of a real wastewater sample for 5, 10, 20, 30, 60, 90, 180, 360 min, 24 h, and 48 h on a laboratory shaker (IKA KS 260 basic). The mixture was then filtered with a glass-fiber filter using 0.45 μm pore-size membranes, and the filtrates were further analyzed. For the adsorption isotherm study, we spiked a real wastewater sample with pre-selected CECs (15 PhACs, 12 pesticides in current use) to obtain diverse initial concentrations of the micropollutants. The selection of CECs to be included in the pre-selected list was done taking into consideration the CECs identified during the previously undertaken studies [7,9,73,74], which involved the analysis of surface and wastewater samples from the region of interest. The influence of the pH levels (6, 7, 8, and the original pH of the wastewater sample) and the adsorbent dose (1.0, 0.5, and 0.2 g/L) was also examined. The pH of the wastewater samples was adjusted using HCl and NaOH. The adsorption capacity was calculated as follows:

q (ng/L) = (C_0_ − C)/m, (2)

where C_0_ (ng/L) is the initial concentration of the organic compound, C (ng/L) is the concentration of the same compound after adsorption, and m (g) is the mass of biochar used per liter of effluent. Were determined the CEC concentrations by high-performance liquid chromatography coupled to triple quadrupole mass spectrometry (UHPLC–MS/MS). For compounds that were not detected or detected in a concentration lower than the method’s limit of quantification (MLOQ) after adsorption, for the calculation of adsorption capacity, we expressed residual concentrations as MLOQ/2 (Table S1).

We also investigated the possibility of multi-stage batch adsorption. The biochar was mixed with a real wastewater sample (1 g/L, original pH) for 45 min. After filtration, the biochar was transferred to a clean and dry Erlenmeyer flask and used again under the same conditions as a fresh aliquot of the wastewater sample. Three consecutive adsorption cycles were performed.

Repeatability for the components’ adsorption was evaluated by conducting three tests under identical conditions (including the same sample of wastewater) with a relative standard deviation (RSD) of 15, 8 and 15% for clarithromycin, propranolol, and linuron, respectively. For all other components, the final concentrations after adsorption tests were lower than MLOQ.

#### 3.3.3. Sample Preparation for UHPLC–MS/MS

We prepared the water samples according to the method described by Gros et al. [75], Petrović et al. [73], and Rakić et al. [76]. Selected CECs were extracted from water samples by solid-phase extraction (SPE) (Oasis HLB cartridges, 6 mL, 200 mg; Waters Corp., Milford, MA, USA) using a Baker vacuum system (J.T. Baker, Deventer, The Netherlands). Briefly, the cartridges were conditioned with 5 mL of methanol (MeOH, J.T. Backer, Deventer, The Netherlands), followed by 5 mL of HPLC water (18.2 MΩ·cm, Milli-Q purification system, Millipore, Molsheim, France). Water samples (i.e., 100 mL effluent) were pre-concentrated on Oasis HLB cartridges. Afterward, the cartridges were washed with 5 mL of HPLC water and dried under vacuum for 3 h to remove excess water. The retained compounds were eluted using methanol (2 × 4 mL). The extracts were fortified with 20 µL of a mixture of internal standards (Table S1) to a final concentration of 40 ng/mL. The extracts were evaporated to dryness at 30 °C under a gentle nitrogen stream and reconstituted in 1 mL of the first gradient of the mobile phase. Before evaporation, internal standards were added to compensate for possible matrix effects (ion suppression or enhancement) and potential losses during evaporation, but not for losses incurred during the extraction process.

#### 3.3.4. Instrumental Analysis

Instrumental analysis of selected CECs in the prepared extracts was performed using high-performance liquid chromatography coupled to triple quadrupole mass spectrometry (UHPLC–MS/MS, Thermo Fisher Scientific, Waltham, MA, USA), based on the method previously described by Rakić et al. [74]. The separation of the analyzed CECs was performed using a Rapid Resolution HD ZORBAX Eclipse Plus C18 column (100 × 2.1 mm, i.d. 1.8 μm, Agilent Technologies, Santa Clara, CA, USA) with a flow rate of 400 µL/min. The column temperature was maintained at 30 °C, while the sample injection volume was 10 μL. The mobile phase consisted of eluent A, containing water/formic acid (Sigma-Aldrich, St. Louis, MO, USA) (99.9:0.1, *v*/*v*) and 5 mM ammonium acetate (Sigma-Aldrich, St. Louis, MO, USA), and eluent B, which consisted only of MeOH. The gradient program was set as follows: 5% of eluent B, increasing to 95% in 4.5 min, rising to 100% in the following 0.1 min, and held for 1.9 min and then returned to the initial condition. The total run time was 13 min. For identification and quantification of CECs we used a triple quadrupole mass spectrometer (MS/MS) TSQ Vantage (Thermo Fisher Scientific, Waltham, MA, USA) equipped with a heated-electrospray ionization probe (HESI-II, Thermo Scientific, Waltham, MA, USA). The parameters of the ion source were set as follows: spray voltage—3.4 kV, vaporizer temperature, 250 °C; sheath gas pressure, 40 arbitrary units; auxiliary gas pressure, 10 arbitrary units; capillary temperature, 270 °C. The target compounds were analyzed in selected reaction monitoring (SRM) mode. All data were processed using Xcalibur 2.1.0 software (Thermo Fisher Scientific, Waltham, MA, USA).

## 4. Conclusions

Within this study, we tested the adsorption characteristics of biochar obtained through the slow pyrolysis of dry raspberry stalks for the removal of CECs from the effluent of the biological treatment of WWTP. The results demonstrated that adsorption occurs in a very short time (around 5 min), enabling the efficient removal of most detected compounds with minimal contact time, which in turn allows for the use of smaller treatment reactors. The smallest tested dose of biochar, 0.2 g/L, is sufficient to achieve high removal efficiencies, and no significant variation in performance was observed across the common pH range for treated municipal wastewater, eliminating the need for pH adjustment and reducing operational costs. Additionally, biochar retained its adsorption performance across three consecutive batch cycles, confirming its potential for reuse. A comparative study showed that raspberry cane-based biochar performance is similar to commercial powdered activated carbon (PAC) in removing a broad spectrum of PhACs and pesticides from complex wastewater matrices.

To the best of our knowledge, this is the first study to investigate biochar produced from raspberry canes for the removal of CECs from real wastewater. What also stands out in this work is that the tests were carried out using real wastewater samples, which are far more complex than synthetic solutions typically used in laboratory studies. Our main objective was to assess the practical performance of biochar in real wastewater and to determine whether its removal efficiency meets the requirements of the new EU Urban Wastewater Treatment Directive (2024) [13], which requires 80% removal of selected micropollutants. These results give confidence that the biochar is applicable in real-world conditions. Furthermore, the study considers the valorization of regional agricultural waste within a circular economy concept, as well as the challenges of waste management, and contributes to the development of cost-effective and environmentally friendly wastewater treatment strategies. Altogether, the obtained results confirm that raspberry cane-based biochar is an effective, low-cost, and sustainable alternative for removing CEC from wastewater, offering both environmental and operational benefits to modern wastewater treatment. However, future research should focus on continuous-flow column studies and breakthrough curve analyses, which are necessary to evaluate performance under realistic hydraulic conditions typical of industrial wastewater treatment, as well as scalability for pilot systems. We should also examine challenges related to regeneration methods and the disposal of spent material. A techno-economic assessment, combined with life-cycle analysis, would help clarify the competitiveness of raspberry-cane biochar relative to powdered activated carbon (PAC) and other commercial adsorbents.

## Figures and Tables

**Table 1:** CECs found in municipal WWTP effluent.

CEC * (ng/L)		Campaign				
	Influent	I	II	III	IV	V
		Effluent	Effluent	Effluent	Effluent	Effluent
Atenolol	0.50	19.4	458	15.3	39.2	54.1
Bezafibrate	<MLOQ	<MLOQ	<MLOQ	<MLOQ	275	<MLOQ
Carbamazepine	161	319	177	726	507	413
Clarithromycin	82.3	75.3	218	115	60.9	46.9
Famotidine	6.23	27.5	<MLOQ	<MLOQ	11.8	18.1
Furosemide	294	<MLOQ	955	<MLOQ	<MLOQ	<MLOQ
HCTZ (hydrochlorthiazide)	574	816	1127	992	803	1459
Losartan	389	<MLOQ	382	<MLOQ	<MLOQ	<MLOQ
Propranolol	<MLOQ	78.1	1767	93.4	61.0	83.5
Ranitidine	<MLOQ	<MLOQ	<MLOQ	<MLOQ	54.6	<MLOQ
Salbutamol	<MLOQ	37.5	<MLOQ	36.1	49.7	47.5
Sotalol	178	310	321	265	589	481
Acetamiprid	<MLOQ	35.6	<MLOQ	35.3	54.1	188
Diazinon	<MLOQ	32.9	<MLOQ	45.9	54.6	66.5
Linuron	<MLOQ	68.7	<MLOQ	58.8	51.5	55.2
Methamidophos	<MLOQ	119	<MLOQ	146	<MLOQ	91.7
Tebuconazole	<MLOQ	49.2	<MLOQ	58.5	98.2	<MLOQ

MLOQ—method limit of quantification. * Standard pharmaceutical nomenclature was used for PhACs.

**Table 2:** Removal efficiencies of CECs by biochar at different effluent pH values (contact time 24 h, dose 1 g/L).

CEC	Campaign I	Removal (%) *			
	C_0_ (ng/L)	pH 6	pH 7	pH 8	Original pH
Atenolol	19.4	100	100	100	100
Carbamazepine	319	100	100	100	100
Clarithromycin	75.3	33.9	30.3	36.2	36.5
Famotidine	27.5	100	100	100	100
HCTZ	816	100	100	100	100
Propranolol	78.1	35.1	37.3	47	38.8
Salbutamol	37.5	100	100	100	100
Sotalol	310	100	100	100	100
Acetamiprid	35.6	100	100	100	100
Diazinon	32.9	100	100	100	100
Linuron	68.7	37.1	34.2	36.2	35.8
Methamidophos	119	100	100	100	100
Tebuconazole	49.2	100	100	100	100

* An efficiency of 100% was achieved when the residual concentration of the compound of interest after adsorption was below the quantification limit of the applied method (MLOQ) (Table S1).

**Table 3:** Removal efficiencies of CECs in WWTP effluent by biochar (contact time 24 h, dose 1 g/L).

CEC	Campaign II C_0_, (ng/L)	Removal (%) *	Campaign III C_0_, (ng/L)	Removal (%) *
Atenolol	458	100	15.3	100
Carbamazepine	177	100	726	100
Clarithromycin	218	43.7	115	48.9
Furosemide	955	100	<MLOQ	/
HCTZ	1127	96.6	992	100
Losartan	382	100	<MLOQ	/
Propranolol	1767	42.3	93.4	52.7
Salbutamol	<MLOQ	/	36.1	100
Sotalol	321	97.7	265	100
Acetamiprid	<MLOQ	/	35.3	100
Diazinon	<MLOQ	/	45.9	100
Linuron	<MLOQ	/	58.8	26.9

* An efficiency of 100% was achieved when the residual concentration of the compound of interest after adsorption was below the quantification limit of the applied method (MLOQ) (Table S1).

**Table 4:** Removal efficiencies of CECs in original and spiked effluent (contact time 24 h, dose 1 g/L).

СEС	Campaign IV	Spiked Effluent	
	C_0_ (ng/L)	Concentration Range (ng/L)	Removal * (%)
Atenolol	39.2	1605–7589	99.5–100
Bezafibrate	275	850–3533	72.6–91.5
Famotidine	11.8	927–4282	100
HCTZ	803	1544–5705	100
Carbamazepine	507	1467–4566	100
Clarithromycin	60.9	927–5787	96.2–100
Propranolol	61	893–5841	91.6–100
Ranitidine	54.6	947–3998	93.8–100
Salbutamol	49.7	1358–6928	97.9–100
Sotalol	589	2052–7552	100
Acetamiprid	54.1	745–3557	93–100
Diazinon	54.6	1499–4638	94.7–100
Methamidophos	˂MLOQ	158–208	100
Linuron	51.5	796–3094	100
Tebuconazol	98.2	1751–4386	100
Acetaminophen	˂MLOQ	1333–4831	100
Diltiazem	˂MLOQ	172–761	80–97
Dimethoate	˂MLOQ	542–2165	100
Ethopropohos	˂MLOQ	1606–5518	100
Furosemide	˂MLOQ	1294–3996	100
Imazalil	˂MLOQ	389–1080	100
Carbofuran	˂MLOQ	205–784	100
Losartan	˂MLOQ	579–2402	93.3–100
Methidation	˂MLOQ	506–2305	100
Omethoate	˂MLOQ	114–306	100
Propioconazole	˂MLOQ	2334–5831	100

* An efficiency of 100% was achieved when the residual concentration of the compound of interest after adsorption was below the quantification limit of the applied method (MLOQ) (Table S1).

**Table 5:** Removal efficiencies of CECs by PAC and biochar (contact time 24 h and 45 min, dose 1 g/L).

СEС	Biochar			PAC		
	Removal * (%)			Removal * (%)		
	C_0_ (ng/L)	24 h	45 min	C_0_ (ng/L)	24 h	45 min
Atenolol	19.4	100	100	39.2	97.4	96.1
Carbamazepine	319.1	100	100	507	100	100
Clarithromycin	75.3	36.6	28.5	60.9	59.1	68.8
Famotidine	27.5	100	100	11.8	100	100
HCTZ	816	100	99.6	803	100	100
Propranolol	78.1	38.8	45.9	61	42.7	47.9
Salbutamol	37.5	100	100	49.7	46.6	45.1
Sotalol	310	100	100	58.9	100	100
Acetamiprid	35.6	100	100	54.1	100	100
Diazonon	32.9	100	100	54.6	100	100
Linuron	68.7	35.8	38.6	51.5	100	100
Methanidophos	119	100	100	15.1	100	100
Tebuconazol	49.2	100	100	98.2	100	100

* An efficiency of 100% was achieved when the residual concentration of the compound of interest after adsorption was below the quantification limit of the applied method (MLOQ) (Table S1).

**Table 6:** Literature data of CEC removal efficiencies by biochars of various origins.

Feedstock	Biochar	CEC	Adsorption			Ref.
			Kinetics	Equilibrium		
			Time	Conditions	Removal (%)	
Pinewood	525 °C 20 min Nonactivated S_BET_ 47.25 m^2^/g	Carbamazepine	48 h	Single solution (0.5–20 ng/mL) Dose: 0.25 g/L, pH 6	70–99	[28]
Raspberry-based biochar	700 °C, 2 h CO_2 activated_ S_BET_ 450 m^2^/g	Carbamazepine	~5 min	WWTP effluent (1467–4566 ng/L) Dose: 1 g/L, Original pH	100	This study
Biosolids (WWTP)	550 °C, 1.5 h Nonactivated S_BET_ 3.98 m^2^/g	Clarithromycin	~15 min	Solution (10^5^ ng/L) (7 antibiotics mixture) Dose: 5–100 g in 1 L, pH 8.33	70–80	[52]
Cattle manure	550 °C, 1.5 h Nonactivated S_BET_ 11.48 m^2^/g		~80 min	Single solution (10^5^ ng/L) (7 antibiotics mixture) Dose: 5–100 g in 1 L, pH 9.96	80–90	
Spent coffee grounds	550 °C 1.5 h Nonactivated S_BET_ 1.53 m^2^/g		~5 min	Single solution (10^5^ ng/L) (7 antibiotics mixture) Dose: 5–100 g in 1 L, pH 8.27	90	
Raspberry-based biochar	700 °C, 2 h CO_2 activated_ S_BET_ 450 m^2^/g	Clarithromycin	~5 min	WWTP effluent (927–5787 ng/L) Dose: 1 g/L, Original pH	96–100	This study
Pineapple leaf non-fibrous material	550 °C, 2 h n.a. S_BET_ 4.65 m^2^/g PV 0.0097 cm^3^/g	Acetamiprid	~200 min	Single solution (10^7^–2·10^8^ ng/L) Dose: 5 g/L, pH 7	84	[45]
Carbonized coconut shell	700 °C, 2 h Nonactivated S_BET_ 434.8 m^2^/g PV 0.174 cm^3^/g	Diazinon	n.a.	Single solution (10^6^ ng/L) Dose: 2·10^6^ ng/L, pH 7, 2 h	>90	[30]
	700 °C, 2 h H_3_PO_4_ S_BET_ 508 m^2^/g PV 0.203 cm^3^/g		n.a.	Single solution (10^6^ ng/L) Dose: 2·10^6^ ng/L, pH 7, 2 h	>90	
	700 °C, 2 h NaOH S_BET_ 405.9 m^2^/g PV 0.162 cm^3^/g		n.a.	Single solution (10^6^ ng/L) Dose: 2·10^6^ ng/L, pH 7, 2 h	>90	
Raspberry-based biochar	700 °C, 2 h CO_2 activated_ S_BET_ 450 m^2^/g	Diazinon	~5 min	WWTP effluent (1499–4638 ng/L) Dose: 1 g/L, Original pH	94.7–100	This study

SBET—BET specific surface area; PV—pore volume.

**Table 7:** Elemental composition of biochar and PAC obtained by EDX.

	C (wt%)	O (wt%)	Mg (wt%)	P (wt%)	K (wt%)	Ca (wt%)	Total
Biochar	60.9	18.9	3.1	1.3	6.5	9.3	100
PAC	97.9	2.1	-	-	-	-	100

**Table 8:** Quality parameters of treated effluent from the WWTP biological treatment process (period 2020–2023).

	Annual Average		ELV [12] (Discharge into Receiving Body)	
Parameter	Value	Removal (%)	Emission Limit Values	Requested Removal (%)
Suspended solids (mg/L)	5–10.1	97–98	35 (mg/L)	90
pH	6.8–8.1	-	6–9	-
COD (mg O_2_/L)	27–36	96	125 (mg/L)	75
BOD_5_ (mg O_2_/L)	3–5.2	98–99	25 (mg/L)	70–90
Total nitrogen (mg/L)	5.7–7.2	89–92	10 mg/L	70–80
Total phosphorus (mg/L)	0.51–1	93–95	1 mg/L	80

COD—Chemical Oxygen Demand. BOD_5_—Biological Oxygen Demand.

## Data Availability

Data is contained within the article or Supplementary Materials.

## Supplementary Materials

The following supporting information can be downloaded at: https://www.mdpi.com/article/10.3390/molecules30244803/s1, Table S1: Validation parameters: instrumental linearity, the method limits of quantification (MLOQ), recoveries, and precision (%RSD); Table S2: Removal efficiencies of nonactivated and activated biochar (1 g/L, 24 h); Table S3: CEC removal efficiencies by biochar and PAC (dose 20 mg/L, 45 min); Table S4. Properties of CECs. References [66,77,78,79,80] are cited in the Supplementary Materials.

## Abbreviations

The following abbreviations are used in this manuscript:

WWTPs	Wastewater treatment plants
CECs	Contaminants of emerging concern
PAC	Powdered activated carbon
PhACs	Pharmaceutically active compounds
PFAS	poly- and perfluoroalkyl substances
AC	Activated carbon
MLOQ	Method limit of quantification
HCTZ	Hydrochlorothiazide
DOC	Dissolved organic carbon
SBET	BET specific surface area
PV	Pore volume
HRSEM	High-resolution scanning electron microscopy
EDX	X-ray energy dispersion
FTIR	Fourier transform infra-ed spectroscopy
BET	Brunauer–Emmett–Teller specific surface
COD_b_	Biodegradable Chemical Oxygen Demand
BOD_5_	Biological Oxygen Demand
UHPLC–MS/MS	high-performance liquid chromatography coupled to triple quadrupole mass spectrometry
