# Supplementary material for: Adsorption Performance Assessment of Agro-Waste-Based Biochar for the Removal of Emerging Pollutants from Municipal WWTP Effluent
Source: Molecules. 2025 Dec 17;30(24):4803. doi: 10.3390/molecules30244803 (PMC12735814; doi:10.3390/molecules30244803)
Supplement: Supplementary file 1 [file molecules-30-04803-s001.zip › molecules-4011942-supplementary.pdf]

Adsorption Performance Assessment of Agro-Waste-Based Biochar for the Removal of Emerging Pollutants from Municipal WWTP Effluent

Dragana Lukić, Vesna Vasić, Jelena Živančev, Igor Antić, Sanja Panić, Mirjana Petronijević and Nataša Đurišić-Mladenović

Supplementary Materials

Investigated CEC

Pharmaceutically active compounds and polar pesticides investigated in this study are presented in Table S1. Table S1 provides the names of the compounds and the most important validation parameters of the applied method (calibration curve linearity, method limits of quantification (MLOQ), recoveries, and precision (%RSD)). The validation parameters confirm that the applied sample preparation method (solid-phase extraction) and instrumental analysis are suitable for quantifying the analytes of interest in the examined matrix. The validation parameters were compared with data available in the literature, where wastewater from treatment plants was used as the matrix for analysis.

Chemicals and reagents

The target method used in this study encompassed pharmacologically active compounds and pesticides currently used (Table S1). High-purity CECs standards were purchased from Sigma Aldrich (St. Louis, MO, USA) or LGC (Augsburg, Germany). LC-MS grade methanol (MeOH), provided by VWR International (Radnor, USA), was used for the preparation of a stock solution with targeted PhACs and pesticides, as well as in solid-phase extraction (SPE) for adsorbent conditioning and elution of CECs. Ultrapure (Milli Q) water (18 MΩ cm), produced by the Advantage system from Millipore (Molsheim, France), was used for sample solution preparation. Quantification was done by using internal calibration curves. The following mass-labelled standards (obtained from HPC Standards GmbH, Cunnersdorf, Germany) were used: Sotalol-D7, Bezafibrate-D6, Carbofuran-D3, Furosemide-D5, Carbamazepine-D10, Acetamiprid-D3, Malathion-D7, Omethoate-D6, Melathion-D7.

Table S1. Validation parameters: instrumental linearity, the method limits of quantification (MLOQ), recoveries, and precision (%RSD)

Compounds	IUPAC Name	CAS number	Molecular formula	Linearity, R^2	MLOQ, ng/L	Recovery, %	Precision (%RSD)	Mass-labelled standards
				Pharmaceutical active compounds				
Atenolol	2-[4-[2-hydroxy-3-(propan-2-ylamino)propoxy]phenyl]acetamide	29122-68-7	$C_{14}H_{22}N_2O_3$	≥0.9900	0.495	90.5	5.5	Sotalol-D7
Acetaminophen	N-(4-hydroxyphenyl)acetamide	103-90-2	$C_8H_9NO_2$	≥0.9900	9.22	84.2	7.4	Sotalol-D7
Bezafibrate	2-[4-[2-[(4 chlorobenzoyl)amino]ethyl]phenoxy]-2-methylpropanoic acid	41859-67-0	$C_{19}H_{20}ClNO_4$	≥0.9900	0.481	78.5	7.2	Bezafibrate-D6
Diltiazem	[(2S,3S)-5-[2-(dimethylamino)ethyl]-2-(4-methoxyphenyl)-4-oxo-2,3-dihydro-1,5-benzothiazepin-3-yl] acetate	42399-41-7	$C_{22}H_{26}N_2O_4S$	≥0.9900	5.61	108	9.3	Carbofuran-D3
Famotidine	3-[[2-(diaminomethylideneamino)-1,3-thiazol-4-yl]methylsulfanyl]-N'-sulfamoylpropanimidamide	76824-35-6	$C_8H_{15}N_7O_2S_3$	≥0.9900	3.22	92.1	7.4	Sotalol-D7
Hydrochlorothiazide (HTCZ)	6-chloro-1,1-dioxo-3,4-dihydro-2H-1lambda6,2,4-benzothiadiazine-7-sulfonamide	58-93-5	$C_7H_8ClN_3O_4S_2$	≥0.9900	2.88	102	3.5	Furosemide-D5
Carbamazepine	benzo[b][1]benzazepine-11-carboxamide	298-46-4	$C_{15}H_{12}N_2O$	≥0.9900	1.20	98.2	8.6	Carbamazepine-D10
Clarithromycin	(3R,4S,5S,6R,7R,9R,11R,12R,13S,14R)-6-[(2S,3R,4S,6R)-4-(dimethylamino)-3-hydroxy-6-methyloxan-2-yl]oxy-14-ethyl-12,13-dihydroxy-4-[(2R,4R,5S,6S)-5-hydroxy-4-	81103-11-9	$C_{38}H_{69}NO_{13}$	≥0.9900	4.25	72.3	10	Carbamazepine-D10

	methoxy-4,6-dimethyloxan-2-yl]oxy-7-methoxy-3,5,7,9,11,13-hexamethyl-oxacyclotetradecane-2,10-dione							
Furosemide	4 4-chloro-2-(furan-2-ylmethylamino)-5-sulfamoylbenzoic acid	54-31-9	$C_{12}H_{11}ClN_2O_5S$	≥0.9900	21.2	64.2	5.9	Furosemide-D5
Losartan	[2-butyl-5-chloro-3-[[4-[2-(2H-tetrazol-5-yl)phenyl]phenyl]methyl]imidazol-4-yl]methanol	114798-26-4	$C_{22}H_{23}ClN_6O$	≥0.9900	10.3	79.8	9.1	Bezafibrate-D6
Ranitidine	(E)-1-N'-[2-[[5-[(dimethylamino)methyl]furan-2-yl]methylsulfanyl]ethyl]-1-N-methyl-2-nitroethene-1,1-diamine	66357-35-5	$C_{13}H_{22}N_4O_3S$	≥0.9900	8.75	87.2	7.5	Sotalol-D7
Salbutamol	4-[2-(tert-butylamino)-1-hydroxyethyl]-2-(hydroxymethyl)phenol	18559-94-9	$C_{13}H_{21}NO_3$	≥0.9900	0.320	93.5	9.3	Sotalol-D7
Sotalol	N-[4-[1-hydroxy-2-(propan-2-ylamino)ethyl]phenyl]methanesulfonamide	3930-20-9	$C_{12}H_{20}N_2O_3S$	≥0.9900	0.220	98.5	10	Sotalol-D7
Propranolol	1-naphthalen-1-yloxy-3-(propan-2-ylamino)propan-2-ol	525-66-6	$C_{16}H_{21}NO_2$	≥0.9900	0.722	78.6	7.1	Carbamazepine-D10
			Pesticides					
Acetamiprid	N-[(6-chloro-3-pyridinyl)methyl]-N-methylethanimidamide	135410-20-7	$C_{10}H_{11}ClN_4$	≥0.9900	11.5	98.6	4.4	Acetamiprid-D3
Carbofuran	(2,2-dimethyl-3H-1-benzofuran-7-yl) N-methylcarbamate	1563-66-2	$C_{12}H_{15}NO_3$	≥0.9900	19.9	101	7.5	Carbofuran-D3
Diazinon	diethoxy-(6-methyl-2-propan-2-ylpyrimidin-4-yl)oxy-sulfanylidene-lambda5-phosphane	333-41-5	$C_{12}H_{21}N_2O_3PS$	≥0.9900	8.25	78.2	8.7	Melathion-D7

Dimethoate	2-dimethoxyphosphinothioylsulfanyl-N-methylacetamide	60-51-5	$C_5H_{12}NO_3PS_2$	≥0.9900	4.95	106	5.2	Acetamiprid-D3
Ethoprophos	1-[ethoxy(propylsulfanyl)phosphoryl]sulfanylpropane	13194-48-4	$C_8H_{19}O_2PS_2$	≥0.9900	13.2	72.3	6.8	Acetamiprid-D3
Methamidophos	[amino(methylsulfanyl)phosphoryl]oxymethane	10265-92-6	$C_2H_8NO_2PS$	≥0.9900	9.25	92.6	11	Acetamiprid-D3
Methidation	3-(dimethoxyphosphinothioylsulfanylmethyl)-5-methoxy-1,3,4-thiadiazol-2-one	950-37-8	$C_6H_{11}N_2O_4PS_3$	≥0.9900	4.63	80.5	9.9	Carbofuran-D3
Omethoate	2-dimethoxyphosphorylsulfanyl-N-methylacetamide	1113-02-6	$C_5H_{12}NO_4PS$	≥0.9900	14.5	117	12	Omethoate-D6
Propiconazole	2-[2-(1-chlorocyclopropyl)-3-(2-chlorophenyl)-2-hydroxypropyl]-1H-1,2,4-triazole-3-thione	60207-90-1	$C_{15}H_{17}Cl_2N_3O_2$	≥0.9900	3.25	63.2	8.8	Melathion-D7
Linuron	3-(3,4-dichlorophenyl)-1-methoxy-1-methylurea	330-55-2	$C_9H_{10}Cl_2N_2O_2$	≥0.9900	4.27	70.2	7.9	Carbofuran-D3
Imazalil	1-[2-(2,4-dichlorophenyl)-2-prop-2-enoxyethyl]imidazole	35554-44-0	$C_{14}H_{14}Cl_2N_2O$	≥0.9900	5.25	78.2	4.4	Carbofuran-D3
Tebuconazole	1-(4-chlorophenyl)-4,4-dimethyl-3-(1,2,4-triazol-1-ylmethyl)pentan-3-ol	107534-96-3	$C_{16}H_{22}ClN_3O$	≥0.9900	22.6	75.2	8.8	Melathion-D7

Table S2. Removal efficiencies of nonactivated and activated biochar (1 g/L, 24 h)

CEC	Removal (%)	
	Nonactivated biochar	Activated biochar
Atenolol	NR	100*
Sotalol	NR	100
Ranitidine	NR	100
HCTZ	NR	100
Propranolol	NR	42.3
Furosemide	75.7	100
Carbamazepine	NR	100
Clarithromycin	NR	43.7
Losartan	100	100

* *An efficiency of 100% was achieved when the residual concentration of the compound of interest after adsorption was below the quantification limit of the applied method*

NR – removed < 20 %

Table S3. CECs removal efficiencies by biochar and PAC (dose 20 mg/L, 45 min)

CEC	Campaign V	Removal (%)*	
	C_0 (ng/l)	Biochar	PAC
Atenolol	54.1	20.5	80.3
Famotidine	18.1	NR	100
Carbamazepine	413	NR	54.4
Clarithromycin	46.9	NR	-
HCTZ	1459	20.1	65.1
Propranolol	83.5	28.1	100
Salbutamol	47.5	NR	27.9
Sotalol	481	NR	66.5
Acetamipride	188	NR	34.6
Diazinon	66.5	NR	-
Linuron	55.2	NR	-
Methamoidophos	91.7	NR	28.8

* *An efficiency of 100% was achieved when the residual concentration of the compound of interest after adsorption was below the quantification limit of the applied method*

NR – removed < 20 %

Table S4. Properties of CECs

	Structure	Molecular Weight (g/mol)	Log Kow	pKa	Length, nm*	Width, nm*	Depth, nm*	Ref.
Atenolol	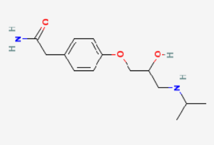	266.34	0.16	9.6	1.215	0.688	0.433	[77]
Bezafibrate	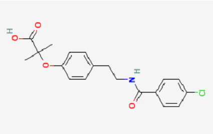	361.8		3.83	1.271	0.876	0.432	[77,78]
Diltiazem	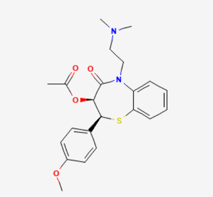	414.5	2.7	8.02	1.230	0.847	0.817	[77]
Famotidine	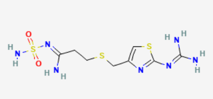	337.5		9.29	1.007	0.707	0.405	[77]
HCTZ	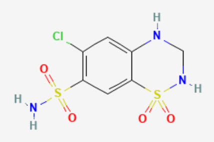	297.7	-0.07	7.9	0.844	0.534	0.339	[77]
Carbamazepine	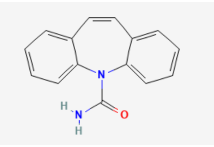	236.27	2.45	pKa1 2.3; pKa 2 13.9	0.911	0.650	0.412	[77,79]
Clarithromycin	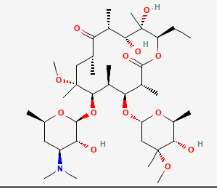	748.0	3.16	8.99	1.183	1.359	0.966	[77]
Propranolol	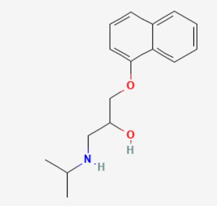	259.34		9.53	0.876	0.679	0.528	[77]
Ranitidine	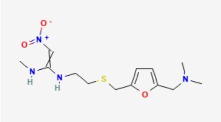	314.41		8.31	1.199	0.807	0.490	[77]
Salbutamol	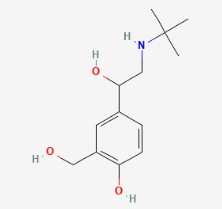	239.31	0.64	10.3	0.982	0.573	0.555	[77]

Sotalol	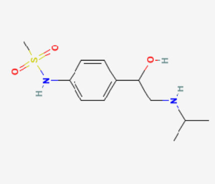	272.37		9.76	1.278	0.523	0.417	[77]
Acetamiprid	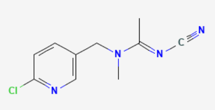	222.67	0.8	0.7	0.881	0.589	0.424	[77]
Diazinon	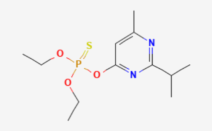	304.35	3.81	2.6	1.034	0.823	0.542	[77]
Methamidophos	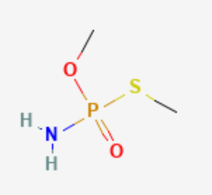	141.13	-0.8		0.582	0.449	0.273	[77]
Linuron	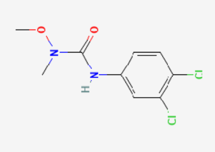	249.09	3.2	12.13	0.994	0.549	0.260	[77,80]
Tebuconazol	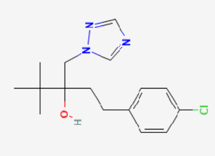	307.82	3.7	5.03	1.056	0.872	0.441	[77,66]

Molecular size descriptors (length, width, and depth) were obtained using 3D conformers in MDL SDF format retrieved from PubChem. The structures were imported into the open-source software Jmol (version 16.3.27) for the calculation of size-related descriptors (Table S4).
